# Gastric Pneumatosis After Accidental Ingestion of Concentrated Hydrogen Peroxide: A Case Report

**DOI:** 10.5811/cpcem.2021.6.52944

**Published:** 2021-10-05

**Authors:** Christine X.Q. Pham, Casey Graves, Michelle Uttaburanont, Karan P. Singh, Maciej Witkos

**Affiliations:** * Rancho Family Medical Group, Temecula, California; † Loma Linda University, Department of Emergency Medicine, Loma Linda, California; ‡ University of California, Riverside, School of Medicine, Riverside, California

**Keywords:** hydrogen peroxide ingestion, gastric pneumatosis, caustic injury, portal venous air, case report

## Abstract

**Introduction:**

Hydrogen peroxide is a common oxidizing agent that if ingested may cause injury to the gastrointestinal tract or embolic events. Although therapy is primarily supportive, gastric perforation is a rare but serious complication of corrosive ingestion that may require surgical treatment.

**Case Report:**

We report the case of a 77-year-old male who presented for nausea and vomiting after accidentally ingesting approximately 150 milliliters of 35% hydrogen peroxide. Computed tomography revealed gastric pneumatosis and extensive portal venous air. The patient was admitted for observation with plans for endoscopy; however, due to the limitations of our small community hospital, he was transferred to a tertiary care center due to concern for a potential gastric perforation.

**Conclusion:**

The presence of portal venous air as a result of peroxide ingestion may be treated conservatively depending on presenting symptoms; however, severe injury such as gastrointestinal perforation may necessitate surgical intervention.

## INTRODUCTION

Hydrogen peroxide is a common oxidizing agent often found in household items such as general-purpose disinfectants, hair dyes, and whitening toothpaste. Higher concentrations of hydrogen peroxide are used as a bleaching agent in the manufacture of paper and textiles, as well as a propellant in rocket fuel. Ingestion of concentrated hydrogen peroxide causes toxicity by one of three mechanisms: corrosive damage; oxygen gas formation; and lipid peroxidation.^1^ There is a growing body of literature regarding hydrogen peroxide ingestion causing injury to the gastrointestinal tract or embolic events. Although therapy is primarily supportive, including the use of hyperbaric oxygen therapy in the presence of gas emboli,^2^ gastric perforation is a rare but serious complication of corrosive ingestion that may require surgical treatment. We present one such case of a patient who developed gastric pneumatosis following ingestion of concentrated hydrogen peroxide.

## CASE REPORT

A 77-year-old male with a past medical history significant for hypertension, stroke, and prostate cancer presented to the emergency department for accidental ingestion with nausea and vomiting. Approximately 90 minutes prior to arrival, the patient had gone to the refrigerator for water and had mistaken a bottle of 35% hydrogen peroxide for water, subsequently ingesting approximately 150 milliliters (mL). The patient immediately felt a burning sensation in his mouth and throat, followed by nausea and vomiting. He reportedly vomited several times, mostly dry heaving, but at one point had a small amount of blood in the vomitus. He otherwise denied any other complaints.

The patient was in no acute distress upon arrival. His blood pressure was elevated at 157/84 millimeters of mercury, respiratory rate 20 breaths per minute, pulse 65 beats per minute, and arterial oxygen saturation was 98% on room air. Complete physical examination was negative for posterior oropharyngeal erythema, abdominal tenderness, rebound, or guarding. Laboratory results were significant for an elevated leukocyte count of 18.9 × 10^3^ thousand per millimeters cubed (K/mm^3^) (reference range: 4.5–11.0 K/mm^3^), hemoglobin of 16.4 grams per deciliter (g/dL) (12.0–16.0 g/dL), creatinine of 1.7 milligrams (mg)/dL (0.5–1.4 mg/dL), and lipase of 844 U/L (73–393 U/L). Initial imaging via computed tomography (CT) of the abdomen and pelvis with contrast revealed gastric pneumatosis extending into the distal esophagus with extensive portal venous air, diverticulosis, multiple left renal cysts, and a 5.8-centimeter (cm) adrenal myolipoma (Image).

Given the patient’s presenting symptoms combined with the CT findings of gastric pneumatosis, the poison center and gastroenterology were consulted. The patient was admitted for observation with plans for an endoscopy; however, upon surgical consultation, he was emergently transferred to a tertiary care hospital due to concerns for a potential perforation and necrosis of the gastric body and distal esophagus, which may have necessitated a total gastrectomy as well as distal esophagectomy. The patient had been placed on a nothing-by-mouth restriction on admission and was hemodynamically stable on intravenous (IV) fluids, pantoprazole IV drip, and piperacillin-tazobactam at time of transfer.

Upon arrival at the tertiary center, the patient reported overall improvement of his abdominal pain and sore mouth. Esophagogastroduodenoscopy performed there confirmed moderate to severe distal esophagitis and severe pangastritis but no evidence of necrosis, linear ulcerations along the lesser curvature, or mild bulbar duodenitis. Gastric biopsy later demonstrated fundic type gastric mucosa with acute inflammation and epithelial erosion. Following his endoscopy, the patient tolerated clear liquids and had his diet advanced to surgical soft. He remained pain-free and was deemed stable for discharge on the third day with recommendations for a repeat endoscopy in two to three months.

**CPC-EM Capsule**

What do we already know about this clinical entity?

*Ingestion of concentrated hydrogen peroxide may result in gastrointestinal, cardiorespiratory, and neurologic effects.*

What makes this presentation of disease reportable?

*Imaging from the computerized tomography scan clearly demonstrates key findings such as gastric pneumatosis and extensive portal venous gas.*

What is the major learning point?

*While management of hydrogen peroxide ingestion consists mainly of supportive care, cases of severe injury may require surgical intervention.*

How might this improve emergency medicine practice?

*Awareness of the clinical manifestations of corrosive ingestion and obtaining prompt imaging can prevent delay in appropriate therapy.*

## DISCUSSION

Hydrogen peroxide is a colorless, odorless liquid used in many household products and can be easily purchased at high concentrations in health food stores. At-home use of hydrogen peroxide has gained popularity because of the purported, and unproven, health benefits aiding in the treatment of rheumatoid arthritis, cancer, human immunodeficiency virus, and Alzheimer’s dementia.^3^ Due to the similarity of its characteristics to water, inappropriate storage of this agent can lead to accidental and potentially serious ingestion. The toxicity of concentrated hydrogen peroxide comes from its ability to damage local tissue and to rapidly decompose into water and oxygen in the presence of the enzyme catalase. One mL of 35% concentrated hydrogen peroxide will liberate 100 mL oxygen. Subsequent generation of large volumes of oxygen in closed body cavities may result in venous or arterial gas embolization, as well as perforation of the hollow viscus.^1^

The most common caustic injury noted on early endoscopy after ingestion is a grade I superficial mucosal injury that often heals spontaneously.^4^ Although ingestion of hydrogen peroxide in lower concentrations is typically nontoxic, it has been reported that consumption of even a small amount can cause portal venous gas embolism and hemorrhagic gastritis.^5^ Ingestion of hydrogen peroxide in higher concentrations is associated with a wide array of effects including gas embolization, moderate to severe gastrointestinal irritation, cardiorespiratory arrest, and cerebral infarct.^1^ In a 10-year retrospective study of 294 cases of high-concentration peroxide ingestion, 13.9% of the patients demonstrated evidence of embolic events.^6^

Management of hydrogen peroxide ingestion depends on the extent of injury, the depth of which can be assessed by endoscopic evaluation. Treatment consists mainly of supportive care, including the use of proton pump inhibitors and H2 antagonists for mild esophageal and gastric symptoms. In patients who develop neurological symptoms such as altered mental status, seizure, and apnea, as the result of systemic gas emboli, hyperbaric oxygen therapy (HBO) has been successfully used with symptom resolution.^7^–^9^ This process involves intermittent inhalation of 100% oxygen under a pressure greater than 1.0 atmospheres absolute (ATA), usually at least 1.4 ATA.^10^ The use of HBO to deliver increased oxygen to body tissues was initially reserved for decompression sickness in deep sea divers but has since been found to be effective as adjunctive therapy for the treatment of acute traumatic wounds, air embolism, gas gangrene, and compartment syndrome.^11^

Additionally, surgical intervention is indicated where there is grade III injury. As there was a strong suspicion for gastric perforation in our patient, an endoscopic examination was not initially performed. Further surgical consultation was deferred to a tertiary care center; however, surgery was ultimately not indicated as the patient was not found to have evidence of esophageal or gastric perforation.

## CONCLUSION

Ingestion of concentrated hydrogen peroxide may result in the presence of gas emboli, gastrointestinal injury, or even death. The presence of portal venous air may be treated conservatively depending on presenting symptoms; however, severe injury such as gastrointestinal perforation may necessitate surgical intervention. Household storage and use of 35% concentrated hydrogen peroxide should be cautioned as there is a need to educate the public on the dangers of corrosives to prevent injury.

## Figures and Tables

**Image:**
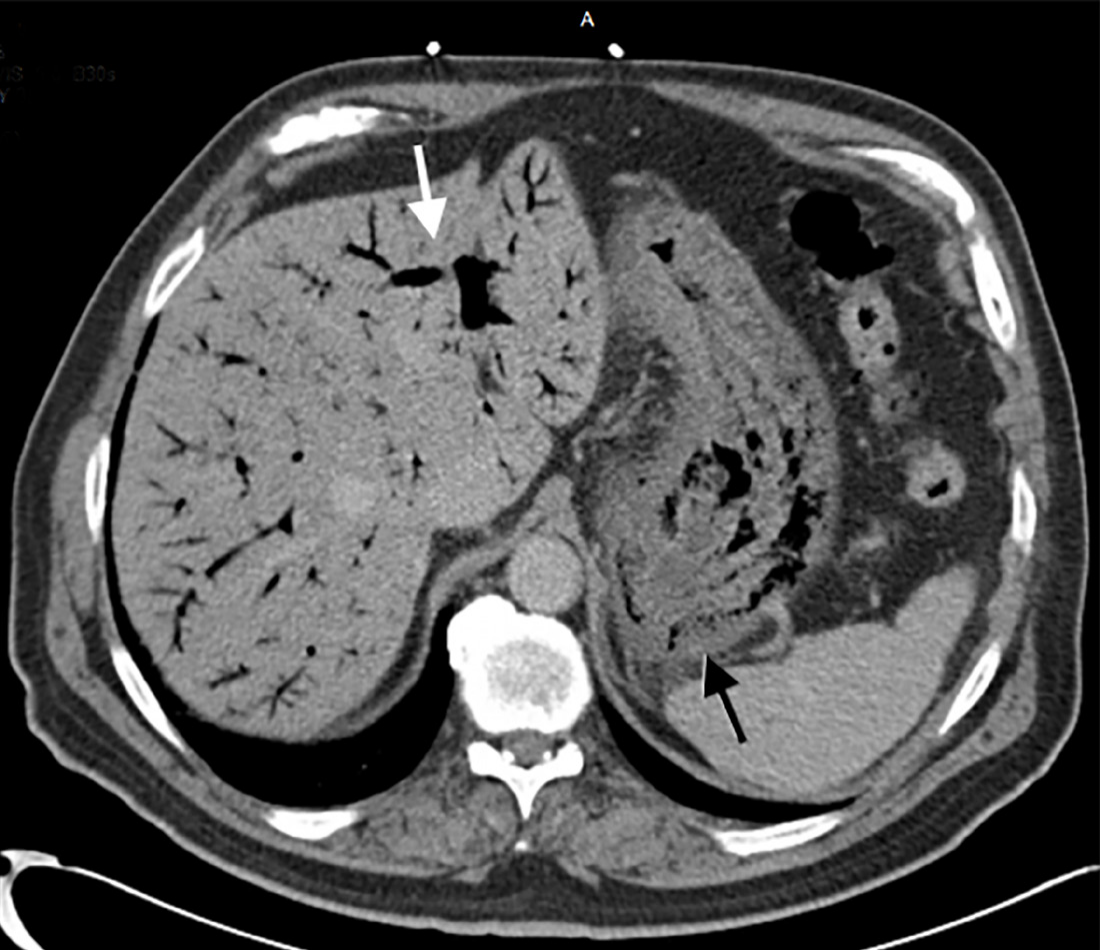
Computed tomography of the abdomen and pelvis without contrast reveals pneumatosis in the walls of the stomach (black arrow) and extensive portal venous gas (white arrow).
